# Analysis of pathogen distribution, clinical features and factors associated with lower respiratory tract multidrug-resistant organism isolation in patients with infectious pneumonia

**DOI:** 10.3389/fmed.2026.1960861

**Published:** 2026-10-30

**Authors:** Jiangbo Li, Bojun Li, Xiang Pei, Bo Li, Dandan Du, Li Zhang

**Affiliations:** Respiratory Intensive Care Unit (RICU), Heping Campus, Tianjin Chest Hospital, Tianjin, China

**Keywords:** factors associated with MDRO isolation, Gram-negative bacteria, infectious pneumonia, multidrug-resistant organism, nomogram

## Abstract

**Objective:**

To investigate pathogen distribution, clinical characteristics, and factors associated with lower respiratory tract multidrug-resistant organism (MDRO) isolation in patients with infectious pneumonia, and to develop an exploratory prediction-oriented nomogram.

**Methods:**

We retrospectively analyzed 117 patients with infectious pneumonia at Tianjin Chest Hospital from September 2024 to September 2025. Bacterial culture was positive in 102 patients. Patients were divided into an MDRO group (*n* = 38) and a non-MDRO group (*n* = 79; 64 with non-MDRO isolates and 15 culture-negative). Pathogen culture, identification, and susceptibility testing were performed. Clinical features were compared, and univariate and binary logistic regression identified factors associated with MDRO isolation. A prediction-oriented nomogram excluding length of RICU stay was developed and internally validated using ROC and calibration curves and 1000 bootstrap resamples.

**Results:**

MDRO isolation occurred in 38 patients: 37.25% (38/102) of culture-positive and 32.48% (38/117) of all enrolled patients. After deduplication, 81 MDRO isolates were identified; 75.31% were Gram-negative and 24.69% Gram-positive. The most common MDROs were Klebsiella pneumoniae (57.89%), Pseudomonas aeruginosa (42.11%), and Acinetobacter baumannii (34.21%); Gram-positive MDROs occurred in 9 patients (23.68%), mostly Staphylococcus aureus (21.05%). The MDRO group had longer RICU stays, higher rates of diabetes, hypoproteinemia, invasive mechanical ventilation, and combined antimicrobial therapy, and lower Glasgow Coma Scale (GCS) scores (all *P* < 0.05). Binary logistic regression identified diabetes (adjusted odds ratio [aOR] = 2.119), invasive mechanical ventilation (aOR=2.849), combined antimicrobial therapy (aOR=2.492), and prolonged RICU stay (aOR=1.137) as associated with MDRO isolation; higher GCS score (aOR=0.674) was associated with lower odds (all *P* < 0.05). The nomogram showed an apparent AUC of 0.862 (95% CI: 0.793–0.931), sensitivity 82.4%, specificity 87.8%, and an optimism-corrected AUC of 0.831 after 1000 bootstrap resamples. Calibration was acceptable (Brier score=0.142, intercept=−0.02, slope=0.88), with some underestimation at intermediate and overestimation at higher predicted probabilities.

**Conclusion:**

MDRO isolation is common in infectious pneumonia, predominantly Gram-negative bacteria. Diabetes, invasive mechanical ventilation, combined antimicrobial therapy, and prolonged RICU stay were associated with MDRO isolation, while higher GCS score was associated with lower odds. An exploratory prediction-oriented nomogram incorporating these factors was constructed but requires prospective validation.

## 1 Introduction

Infectious pneumonia, a respiratory disease that represents a major global threat to human health, continues to show persistently high incidence and mortality rates (1). The extensive use of broad-spectrum antibiotics has made multidrug-resistant organism (MDRO) infections a significant challenge and critical issue in clinical management. These infections not only substantially elevate patients’ hospitalization costs but also serve as a primary contributor to the failure of anti-infection therapy (2, 3). MDRO isolation is often associated with poor response to conventional treatment and persistent recurrence of the condition, severely affecting the recovery process of patients and may even precipitate severe adverse clinical events, including sepsis and multiple organ dysfunction (4).

The occurrence of MDRO infections is not random but is a complex outcome resulting from the combined effects of the host's immune status, underlying diseases, invasive procedures, and previous medication history. Previous studies have mostly focused on single-time-point pathogen investigations and lacked dynamic examinations of the changes in pathogen distribution and their associations with clinical outcomes (5, 6). Recently, as the paradigm of precision medicine has continued to evolve, risk stratification management for specific populations has become a new trend in infection control. By clarifying the composition of MDROs and their resistance spectra, epidemiological evidence can be provided for empirical anti-infection treatment (7). The key to identifying patients at higher odds of MDRO isolation lies in screening reliable early warning indicators. Studies have shown that MDRO colonization or infection is closely related to hospital stay duration, antibiotic exposure history, RICU admission history, and immunosuppression factors (8, 9). However, longitudinal dynamic studies on secondary MDRO infections in patients with infectious pneumonia are still relatively scarce, and the comprehensive assessment of the specific clinical outcomes caused by them is not yet sufficient.

Based on this, this study retrospectively analyzed the clinical data of patients diagnosed with infectious pneumonia, systematically investigated the distribution characteristics of pathogenic bacteria of MDROs, deeply analyzed their impact on the clinical features of patients, and screened out key factors associated with lower respiratory tract MDRO isolation in patients with infectious pneumonia. The objective was to furnish a scientific foundation for the early detection of high-risk patients, the optimization of anti-infection strategies, and the improvement of patient prognosis.

## 2 Materials and methods

### 2.1 Research subjects

A total of 128 patients with clinically suspected infectious pneumonia admitted to Tianjin Chest Hospital between September 2024 and September 2025 were assessed for eligibility. Of these, 11 patients were excluded for the following reasons: immunodeficiency or malignancy (*n* = 5), previous pulmonary surgery (*n* = 1), severe multiple organ failure or pulmonary tuberculosis (*n* = 3), and incomplete clinical data or lost to follow-up (*n* = 2). Finally, 117 patients were enrolled. Bacterial culture was positive in 102 patients, and 15 patients were bacterial culture-negative. The full analysis set included all 117 patients. According to the presence or absence of lower respiratory tract MDRO isolation, they were assigned to an MDRO group (*n* = 38) and a non-MDRO group (*n* = 79). The non-MDRO group included 64 patients with non-MDRO bacterial isolates and 15 bacterial culture-negative patients. The full analysis set was used because the retrospective association model is intended for patients with clinically suspected infectious pneumonia, including those with negative cultures. This research received approval from the Ethics Committee of Tianjin Chest Hospital (Approval Number: 2026LW-044) (Figure 1).

**Figure 1:**
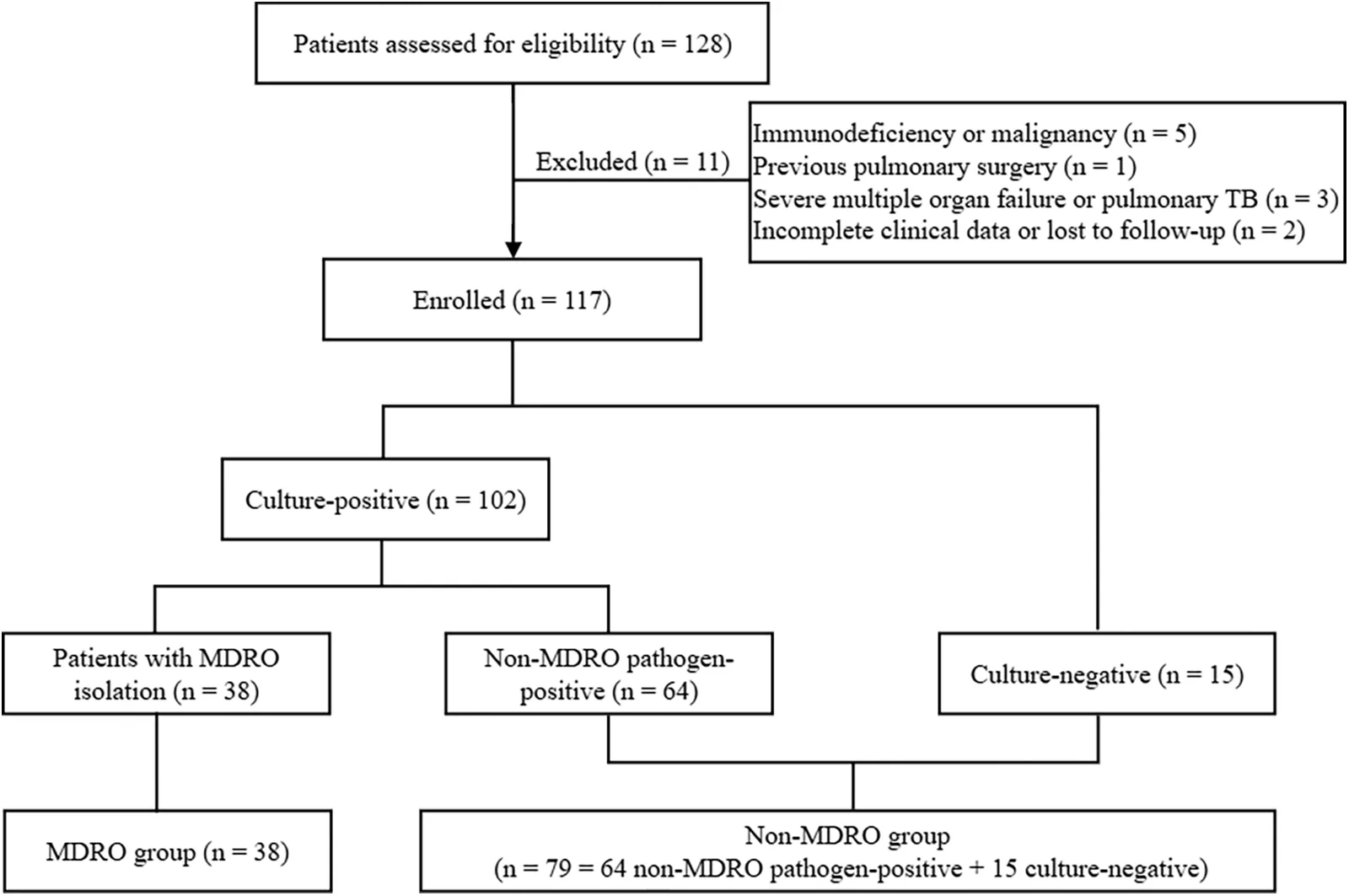
Flow diagram of patient selection.

Pneumonia was classified as community-acquired pneumonia (CAP), hospital-acquired pneumonia (HAP), or ventilator-associated pneumonia (VAP) based on the timing of onset. CAP was defined as pneumonia diagnosed at admission or within 48 h after admission. HAP was defined as pneumonia occurring more than 48 h after admission. VAP was defined as pneumonia occurring more than 48 h after endotracheal intubation. Viral pneumonia was defined as pneumonia with a positive viral test and no evidence of bacterial coinfection at the time of diagnosis. Bacterial pneumonia was defined as pneumonia with a positive bacterial culture or other microbiological evidence of bacterial infection. Mixed pneumonia was defined as pneumonia with both viral and bacterial pathogens identified at the time of diagnosis. In this retrospective cohort, pneumonia was recorded as bacterial or viral based on clinical and microbiological findings at the initial diagnosis; no patient met the definition of mixed pneumonia at the initial diagnosis. Among the 38 patients with MDRO isolation, 30 had bacterial pneumonia at the initial diagnosis and 8 had viral pneumonia with secondary bacterial MDRO isolation. These 8 patients were retained in the viral pneumonia category and were not reclassified as mixed pneumonia, because bacterial coinfection was not documented at the initial diagnosis. MDRO isolation in these 8 patients was considered a secondary bacterial infection, not isolated viral pneumonia. Because of incomplete retrospective records, we could not further classify all patients into CAP, HAP, or VAP.

MDRO isolation criteria: The patient fulfilled the clinical diagnostic criteria for infectious pneumonia, and a pathogenic bacterium isolated from a lower respiratory tract specimen met the standardized definition of multidrug-resistant organisms (MDRO) proposed by the European Centre for Disease Prevention and Control (ECDC) and the US Centers for Disease Control and Prevention (CDC) (10). The American Thoracic Society (ATS)/Infectious Diseases Society of America (IDSA) criteria (11) were used to support the clinical diagnosis of pneumonia. Isolation of an MDRO from a lower respiratory tract specimen was not considered equivalent to MDRO pneumonia or MDRO infection; the primary outcome was therefore defined as lower respiratory tract MDRO isolation.

Inclusion criteria: (1) Age of 18 years or older; (2) Fulfillment of the diagnostic criteria for infectious pneumonia as stipulated in “Internal Medicine”; (3) Completed pathogen testing after admission; (4) Availability of complete clinical records.

Exclusion criteria: (1) Presence of immunodeficiency disorders or active malignancies; (2) Previous history of pulmonary surgery; (3) Complicated with severe multiple organ failure or pulmonary tuberculosis. Patients with severe multiple organ failure were excluded because they often had unstable hemodynamics and incomplete clinical data, which could affect the reliability of pathogen testing and data collection. However, this exclusion may introduce selection bias, as these patients are at high risk for MDRO isolation. (4) Incomplete clinical data or lost to follow-up.

### 2.2 Pathogen culture and identification

For those who can cough up phlegm independently, clean the mouth with normal saline three times and then collect deep phlegm samples; for those who cannot cough up phlegm independently, collect phlegm specimens from the lower respiratory tract using a disposable sterile suction tube through an artificial airway. Specimens were required to be sent for examination within 2 h. Sputum or endotracheal aspirate specimens were collected when pneumonia was clinically suspected, either at admission or during hospitalization. The exact timing of specimen collection relative to admission was not uniformly recorded in this retrospective cohort. The specimens were first subjected to smear microscopic examination. The qualifying threshold is when there are more than 25 white blood cells and fewer than 10 squamous epithelial cells under low-power microscopy. After being inoculated onto the culture medium, the qualifying specimens are cultivated for 48 h at 37 °C. Pathogen identification and antimicrobial susceptibility testing were performed according to the routine laboratory protocol and the manufacturer's instructions. The strains are identified using the VITEK 2 Compact completely automatic microbial identification and drug sensitivity system (Biomerieux, France), and the drug sensitivity data are ascertained using the minimum inhibitory concentration (MIC) approach. Escherichia coli [American Type Culture Collection (ATCC) 25922], Klebsiella pneumoniae (ATCC 700603), Pseudomonas aeruginosa (ATCC 27853), Acinetobacter baumannii (ATCC 19606), Staphylococcus aureus (ATCC 25923), Streptococcus pneumoniae (ATCC 96919), Staphylococcus epidermidis (ATCC 14990), and Enterococcus faecalis (ATCC 29212) are among the quality control strains. Susceptibility testing was performed using the VITEK 2 Compact system according to the manufacturer's instructions. MDR was defined according to the standardized international terminology proposed by ECDC/CDC. Intermediate results were classified as non-susceptible. Because this was a retrospective study, the specific Clinical and Laboratory Standards Institute (CLSI) or European Committee on Antimicrobial Susceptibility Testing (EUCAST) edition and year, the antimicrobial categories used to define MDR, and additional confirmation tests were not recorded in the laboratory database. Intrinsic resistance was not specifically excluded when classifying isolates as MDR because the antimicrobial categories used for MDR classification were not recorded in the retrospective laboratory database. Therefore, detailed resistance profiles and confirmation of resistance phenotypes were not available. Because isolation of an MDRO from a respiratory specimen does not by itself confirm pneumonia, and because colonization and contamination could not be completely distinguished from true infection in this retrospective study, the primary outcome was defined as lower respiratory tract MDRO isolation rather than MDRO pneumonia. Bronchoalveolar lavage fluid (BALF) was not routinely collected in this retrospective cohort; therefore, sputum or endotracheal aspirate cultures were used. Only qualified specimens were included, and colonization and contamination were excluded as far as possible using specimen quality, predominant growth, and clinical correlation.

To avoid overrepresentation of repeated isolates from the same patient, a deduplication rule was applied. For patient-level analysis, only the first isolate of each species per patient per pneumonia episode was included. For isolate-level analysis (e.g., Table 1), all deduplicated isolates were counted; repeated isolates of the same species from the same patient were excluded, while different species from the same patient were counted separately. Polymicrobial cultures were recorded.

**Table 1:** Types and proportions of MDRO pathogens in 38 patients with MDRO isolation.

Types of pathogenic bacteria	Patients, *n* (%)	Isolates, *n* (%)
Gram-negative bacteria	30 (78.95)	61 (75.31)
Klebsiella pneumoniae	22 (57.89)	22 (27.16)
Pseudomonas aeruginosa	16 (42.11)	16 (19.75)
Acinetobacter baumannii	13 (34.21)	13 (16.05)
Escherichia coli	7 (18.42)	7 (8.64)
Others	3 (7.89)	3 (3.70)
Gram-positive bacteria	9 (23.68)	20 (24.69)
Staphylococcus aureus	8 (21.05)	8 (9.88)
Staphylococcus epidermidis	4 (10.53)	4 (4.94)
Enterococcus faecalis	3 (7.89)	3 (3.70)
Streptococcus pneumoniae	3 (7.89)	3 (3.70)
Others	2 (5.26)	2 (2.47)

Patient-level data are based on the first isolate of each species per patient per pneumonia episode. Because some patients had multiple pathogens, the sum of patient counts may exceed 38. Isolate-level data are after species-level deduplication (*n* = 81); repeated isolates of the same species from the same patient were excluded, while different species from the same patient were counted separately. One patient had both Gram-negative and Gram-positive MDRO isolates.

### 2.3 Observation indicators

(1) Distribution of pathogenic bacteria: A statistical analysis was conducted to determine the types and proportions of pathogenic bacteria present in the affected patients. The types of pathogenic bacteria include Gram-negative bacteria and Gram-positive bacteria.
(2) Clinical features: Age, gender, body mass index (BMI), smoking history, drinking history, underlying diseases (diabetes, hypertension, hypoproteinemia), educational level (junior high school or below, college or above), whether the disease occurred in winter, invasive mechanical ventilation, combined antimicrobial therapy, Glasgow Coma Scale (GCS) score, length of stay in the respiratory intensive care unit (RICU), type of infectious pneumonia (bacterial, viral), symptoms (fever, cough, vomiting), respiratory failure, arteriovenous catheterization, radiotherapy. Radiotherapy was recorded as a history of radiotherapy. As active malignancy was an exclusion criterion, no patient had an active malignancy. The specific indication for radiotherapy was not available in the retrospective database. Among them, the GCS score indicates the degree of coma of the patient, which consists of three elements: limb movement, linguistic response, and eye opening reaction. The patient's consciousness issue is more severe if the score is lower, with a total of 15 points (12). GCS, invasive mechanical ventilation, and combined antimicrobial therapy were recorded at the time of RICU admission. Length of RICU stay was defined as the total duration from RICU admission to discharge or transfer from the RICU. It reflects the overall RICU course, was available for all patients, and was not available at RICU admission. Therefore, it was treated as a factor associated with MDRO isolation, not as an early predictor available at RICU admission. Respiratory failure and hypoproteinemia were assessed during hospitalization based on clinical needs. Their exact timing relative to admission or MDRO detection was not standardized. Data on previous antimicrobial exposure, recent hospitalization, transfer from another healthcare facility, previous MDRO colonization or infection, chronic respiratory disease, immunosuppressive treatment, duration of ventilation and device use, and APACHE II or SOFA scores were not routinely collected in this retrospective cohort. Whether patients were sedated or intubated at the time of GCS assessment was not routinely recorded in this retrospective cohort. In intubated patients, the verbal component was scored as 1 (no verbal response), consistent with routine clinical practice.
(3) Analysis of factors associated with MDRO isolation: To investigate factors associated with lower respiratory tract MDRO isolation in patients with infectious pneumonia, a binary logistic regression analysis was performed using variables that differed between the MDRO group and the non-MDRO group as independent variables and MDRO isolation as the dependent variable (yes = 1, no = 0). These variables were interpreted as factors associated with MDRO isolation, rather than as causal factors.

### 2.4 Statistical analysis

SPSS 26.0 was used to statistically evaluate all of the data. Continuous data are presented as mean ± SD and compared using the t-test; categorical data are presented as *n* (%) and compared using the *χ*^2^ test. The full analysis set included 117 patients (38 MDRO and 79 non-MDRO). Binary logistic regression was used to identify factors associated with MDRO isolation; aORs and 95% CIs are shown in a forest plot. The multivariable association model is reported in Table 4. A separate prediction-oriented nomogram excluded length of RICU stay and included diabetes, invasive mechanical ventilation, combined antimicrobial therapy, and GCS score. ROC and calibration curves were used to assess apparent performance. Variable selection was based on clinical relevance and univariate analysis (*P* < 0.05), followed by LASSO regression with 10-fold cross-validation and then multivariable binary logistic regression, without additional stepwise selection. GCS score and length of RICU stay were modelled as continuous variables, and binary variables were coded as 0 or 1. Cross-validation was used only for LASSO variable selection, not for model validation. Of 21 candidate variables, 6 entered LASSO regression and 5 were retained in the multivariable association model (EPV ≈ 7.6); the prediction-oriented nomogram contained 4 predictors (EPV ≈ 9.5). Both models are exploratory. VIF < 5 and tolerance >0.2 were considered acceptable, and *P* < 0.05 was considered statistically significant. Internal validation was performed using 1000 bootstrap resamples, and optimism-corrected performance was calculated with the rms package in R.

## 3 Results

### 3.1 Types and proportions of pathogenic bacteria in the MDRO group

Among the 117 patients in this study, bacterial culture was positive in 102 patients. MDRO was detected in 38 patients, accounting for 37.25% (38/102) of culture-positive patients and 32.48% (38/117) of all enrolled patients. Among the 38 patients with MDRO isolation, 81 MDRO isolates were identified after species-level deduplication. These represented distinct species from the same patient; repeated isolates of the same species from the same patient were excluded, while different species from the same patient were counted separately. At the patient level, the most common MDROs were Klebsiella pneumoniae (22, 57.89%), Pseudomonas aeruginosa (16, 42.11%), and Acinetobacter baumannii (13, 34.21%). Gram-positive MDROs were detected in 9 patients (23.68%), most commonly Staphylococcus aureus (8, 21.05%). Gram-negative bacteria were the predominant bacterial group, totaling 61 isolates (75.31%), and 20 isolates (24.69%) were Gram-positive bacteria. The detailed composition is shown in Table 1. Among the 38 patients with MDRO isolation, 30 had bacterial pneumonia and 8 had viral pneumonia with secondary bacterial MDRO isolation. Thus, all 38 patients had bacterial MDRO isolation, while 8 also had viral pneumonia. MDRO isolation in these 8 patients was considered secondary bacterial infection, not isolated viral pneumonia.

### 3.2 Comparison of general data between the MDRO group and the non-MDRO group

An aggregate of 117 patients were split into two groups: 38 cases in the MDRO group and 79 cases in the non-MDRO group. According to Table 2, there were no statistically significant differences between the two groups in terms of age, gender, BMI, smoking and drinking histories, educational attainment, or the percentage of winter onset (*P* > 0.05).

**Table 2:** Comparison of general data and clinical characteristics between the MDRO group and the non-MDRO group (full analysis set, *n* = 117).

Indicators	MDRO group (*n* = 38)	non-MDRO group (*n* = 79)	*χ*^2^/t	P
Age (year, $\bar{x}\pm s$)	67.32 ± 9.45	65.18 ± 10.27	1.124	0.263
Gender [n (%)]			0.050	0.822
Male	22 (57.89)	44 (55.70)		
Female	16 (42.11)	35 (44.30)		
BMI (kg/m^2^, $\bar{x}\pm s$)	22.17 ± 3.06	22.85 ± 3.41	1.043	0.299
Smoking history [n (%)]			0.497	0.481
Yes	19 (50.00)	34 (43.04)		
No	19 (50.00)	45 (56.96)		
Drinking history [n (%)]			0.176	0.675
Yes	11 (28.95)	20 (25.32)		
No	27 (71.05)	59 (74.68)		
Educational level [n (%)]			0.277	0.599
Junior high school or below	25 (65.79)	48 (60.76)		
College or above	13 (34.21)	31 (39.24)		
Winter onset of the disease [n (%)]			0.116	0.733
Yes	21 (55.26)	41 (51.90)		
No	17 (44.74)	38 (48.10)		
Diabetes [n (%)]			7.215	0.007
Yes	23 (60.53)	27 (34.18)		
No	15 (39.47)	52 (65.82)		
Hypertension [n (%)]			0.314	0.575
Yes	17 (44.74)	31 (39.24)		
No	21 (55.26)	48 (60.76)		
Hypoproteinemia [n (%)]			8.062	0.005
Yes	26 (68.42)	32 (40.51)		
No	12 (31.58)	47 (59.49)		
Invasive mechanical ventilation [n (%)]			9.683	0.002
Yes	24 (63.16)	26 (32.91)		
No	14 (36.84)	53 (67.09)		
Combined antimicrobial therapy [n (%)]			8.746	0.003
Yes	27 (71.05)	33 (41.77)		
No	11 (28.95)	46 (58.23)		
Arteriovenous catheterization [n (%)]			0.333	0.564
Yes	19 (50.00)	35 (44.30)		
No	19 (50.00)	44 (55.70)		
Respiratory failure [n (%)]			0.355	0.551
Yes	21 (55.26)	39 (49.37)		
No	17 (44.74)	40 (50.63)		
GCS score (score, $\bar{x}\pm s$)	9.26 ± 2.14	12.73 ± 1.86	8.993	<0.001
length of RICU stay (d, $\bar{x}\pm s$)	12.53 ± 3.84	4.29 ± 1.47	16.750	<0.001
Pneumonia category [n (%)]			11.701	0.003
Bacterial	30 (78.95)	64 (81.01)		
Viral	8 (21.05)	4 (5.06)		
Bacterial culture-negative/unknown	0 (0.00)	11 (13.92)		
Fever [n (%)]			0.213	0.644
Yes	33 (86.84)	66 (83.54)		
No	5 (13.16)	13 (16.46)		
Cough [n (%)]			0.048	0.827
Yes	36 (94.74)	74 (93.67)		
No	2 (5.26)	5 (6.33)		
Vomiting [n (%)]			0.185	0.667
Yes	7 (18.42)	12 (15.19)		
No	31 (81.58)	67 (84.81)		
Radiotherapy [n (%)]			0.084	0.772
Yes	4 (10.53)	7 (8.86)		
No	34 (89.47)	72 (91.14)		

Non-MDRO group (*n* = 79): 64 patients with non-MDRO bacterial isolates plus 15 bacterial culture-negative patients. The 15 bacterial culture-negative patients were classified as viral pneumonia only when a viral test was positive and bacterial coinfection was absent; otherwise, they were bacterial culture-negative/unknown. Of the 38 MDRO patients, 30 had bacterial pneumonia and 8 had viral pneumonia with secondary bacterial MDRO isolation, classified by initial diagnosis as viral, not mixed, pneumonia.

### 3.3 Comparison of clinical characteristics between the MDRO group and the non-MDRO group

As indicated in Table 2, statistically significant differences existed between the two groups in relation to diabetes, hypoproteinemia, invasive mechanical ventilation, combined antimicrobial therapy, length of RICU stay, and GCS score (*P* < 0.05); pneumonia classification differed between groups (*P* = 0.003), mainly because all bacterial culture-negative/unknown patients were confined to the non-MDRO group by definition; no statistically significant differences were detected for fever, cough, vomiting, or radiotherapy (*P* > 0.05).

### 3.4 Screening of factors associated with MDRO isolation

In the single-factor analysis, six indicators with *P*-values less than 0.05 (diabetes, hypoproteinemia, invasive mechanical ventilation, combined antimicrobial therapy, length of RICU stay, GCS score) were selected as the candidate variables for LASSO regression analysis. Through 10-fold cross-validation, the optimal regularization parameter *λ* (λ.min) was determined, and the corresponding model had the smallest binomial deviance. When *λ* was set to *λ*.min, the coefficients of five variables were not zero, and the final model selected five variables, including diabetes, invasive mechanical ventilation, combined antimicrobial therapy, length of RICU stay, GCS score, as shown in Figures 2A,B.

**Figure 2:**
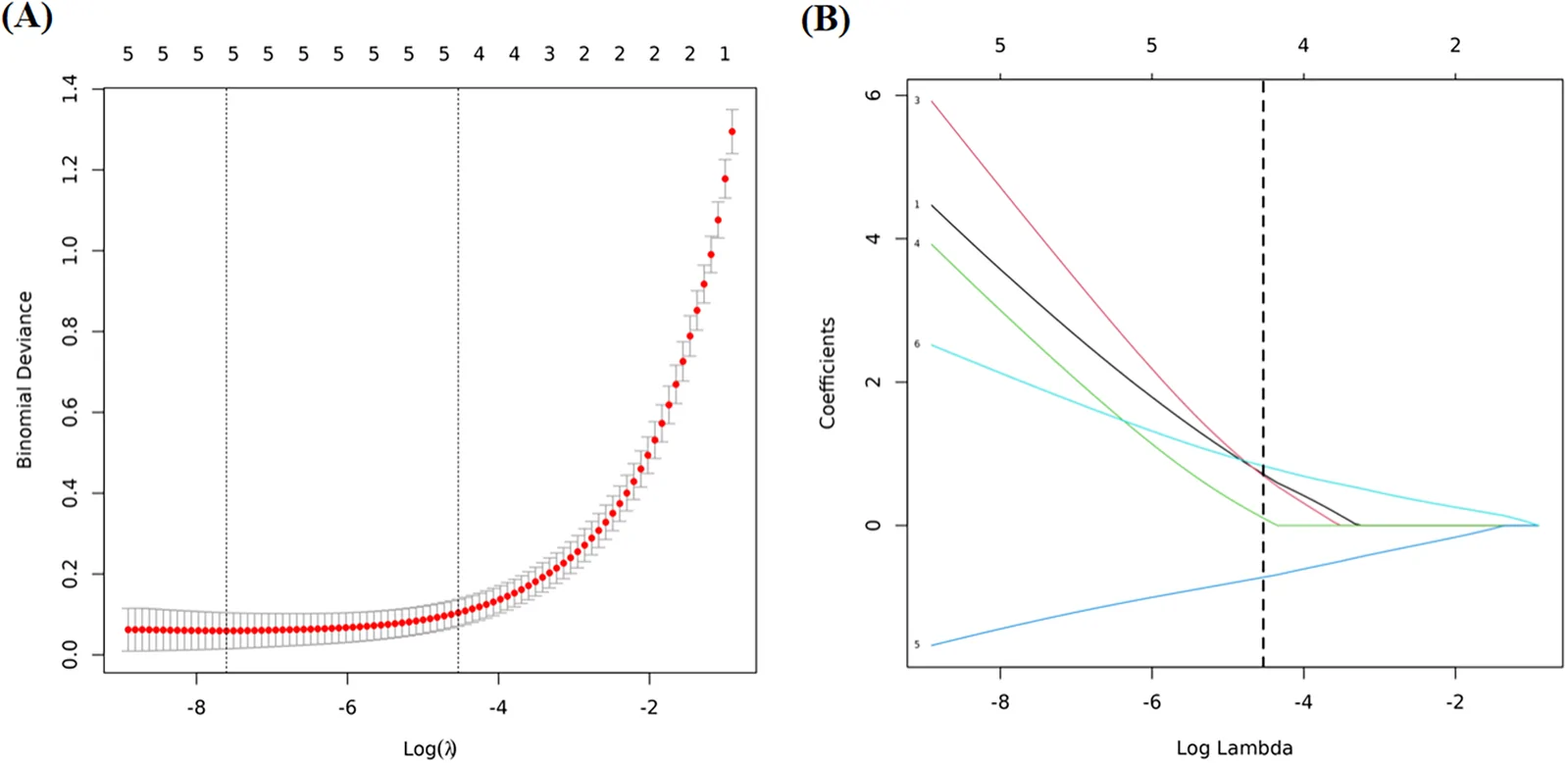
Using the 10-fold cross-validation method, the LASSO regression model was employed to choose the most appropriate features. **(A)** The binomial deviance curve versus log(lambda). **(B)** The coefficient paths versus log(lambda).

### 3.5 Logistic regression analysis of factors associated with MDRO isolation

A multicollinearity test was conducted on variables such as diabetes, invasive mechanical ventilation, combined antimicrobial therapy, length of RICU stay, and GCS score prior to performing the logistic regression analysis in order to prevent the results from being biased due to the high correlation among the independent variables. Measurements were made using the variance inflation factor (VIF) and tolerance. Table 3 displays the outcomes. According to the test results, there was no significant multicollinearity issue among the independent variables since all of the VIF values were less than 5 and the tolerance was higher than 0.2. As a result, the logistic regression analysis that followed included every variable. A univariate logistic regression analysis was performed using all of the observation indicators as independent variables and the occurrence of MDRO isolation as the dependent variable. The findings indicated that a higher GCS score was associated with lower odds of MDRO isolation (*P* < 0.05), while diabetes, invasive mechanical ventilation, combined antimicrobial therapy, and prolonged RICU stay were associated with higher odds (*P* < 0.05). Variables selected by LASSO regression were entered into the multivariable logistic regression model. No further stepwise selection was performed. As indicated in Table 4, four factors associated with MDRO isolation and one factor associated with lower odds were ultimately identified.

**Table 3:** Results of collinearity test for independent variables.

Indicators	Tolerance	VIF
Diabetes	0.786	1.272
Invasive mechanical ventilation	0.693	1.443
Combined antimicrobial therapy	0.715	1.399
length of RICU stay	0.802	1.247
GCS Score	0.764	1.309

### 3.6 Forest plot results of factors associated with MDRO isolation

A forest plot was drawn using the aOR values and 95% CI of multiple factors (Figure 3). The *x*-axis stands for the aOR values, the *y*-axis stands for the various influencing factors, and the vertical line stands for the aOR = 1 null line. The horizontal lines for diabetes, invasive mechanical ventilation, combined antimicrobial therapy, and prolonged RICU stay all completely lie to the right of the null line, suggesting that these are factors associated with MDRO isolation; the GCS score horizontal line is to the left of the null line, indicating a factor associated with lower odds, which is consistent with the results of the regression analysis.

**Figure 3:**
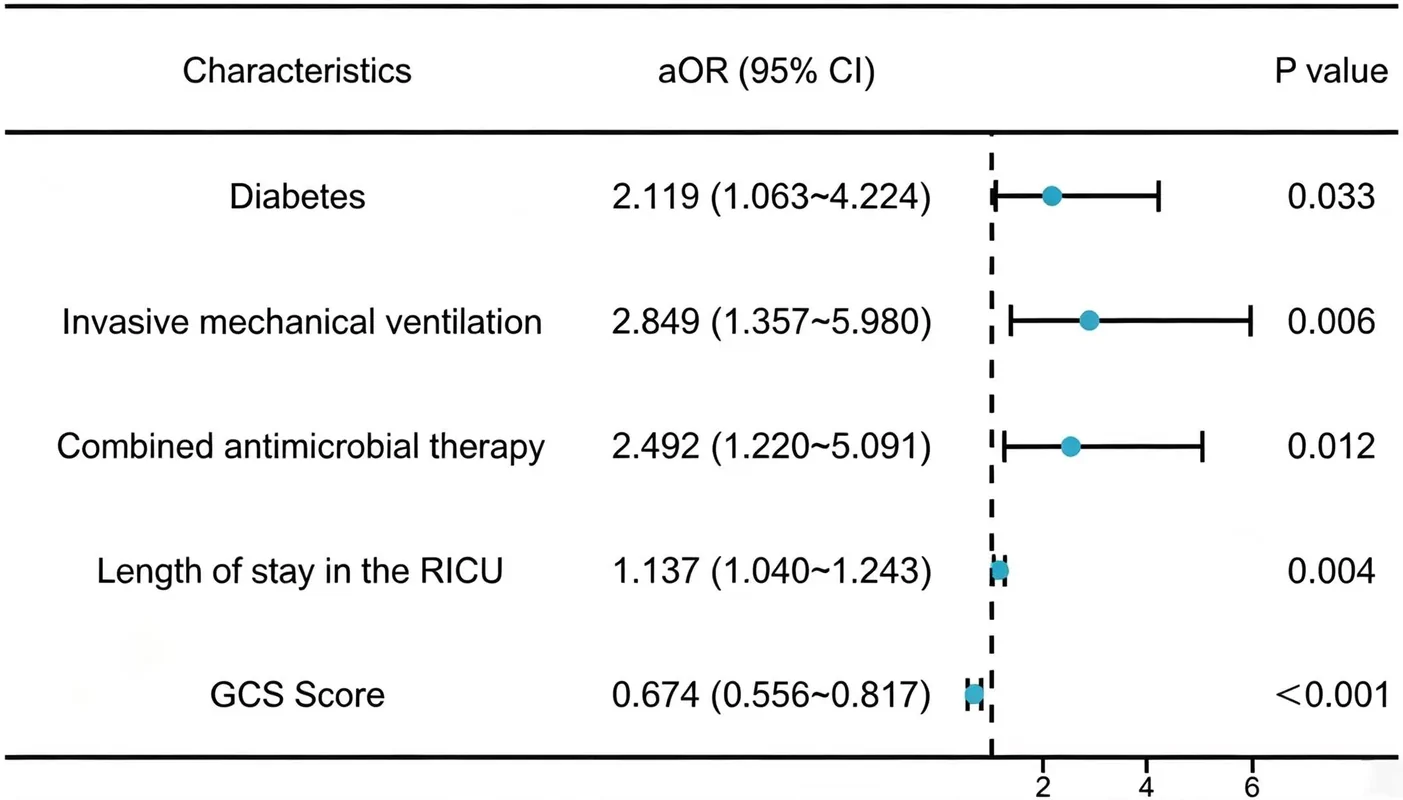
Adjusted odds ratios (aORs) and 95% CIs for independent factors associated with MDRO isolation.

### 3.7 Construction of a prediction-oriented nomogram for MDRO isolation

Figure 4 shows an exploratory, prediction-oriented nomogram for lower respiratory tract MDRO isolation. Because length of RICU stay is known only after the RICU course, it cannot be used for prediction at RICU admission and was therefore excluded from the nomogram. It remains reported in Table 2 and in the univariate analysis as a clinical association variable. The final nomogram includes diabetes, invasive mechanical ventilation, combined antimicrobial therapy, and GCS score. GCS is reverse-scored (15 = 0 points; 3 = 100 points). The linear predictor is:

**Figure 4:**
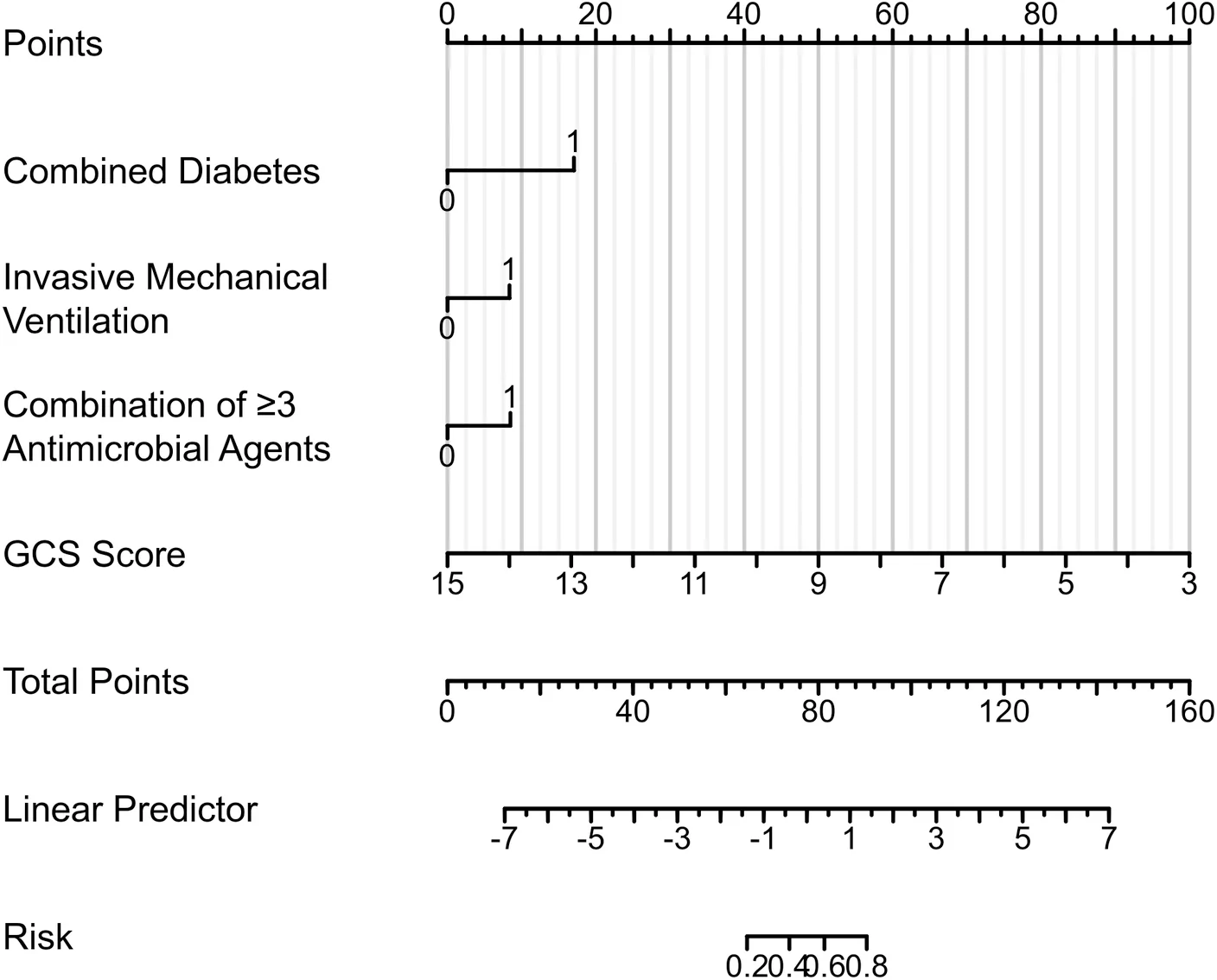
Prediction-oriented nomogram for the probability of lower respiratory tract MDRO isolation. The nomogram includes diabetes, invasive mechanical ventilation, combined antimicrobial therapy, and GCS score; length of RICU stay was excluded. The GCS axis ranges from 3 to 15, the full clinical range; the nomogram should not be applied outside this range. The linear predictor axis is derived from the final prediction-oriented logistic regression model.

LP = 1.711 + 0.751 × diabetes + 1.047 × invasive mechanical ventilation + 0.913 × combined antimicrobial therapy−0.394 × GCS score.

Predicted probability = 1/(1 + exp(−LP)). The nomogram is exploratory and requires internal and external validation before clinical use.

### 3.8 Apparent performance of the prediction-oriented nomogram

The apparent performance of the prediction-oriented nomogram was assessed in the development cohort. The ROC curve yielded an area under the curve (AUC) of 0.862 (95% CI: 0.793–0.931); the sensitivity and specificity corresponding to the Youden index were 82.4% and 87.8%, respectively (Figure 5A). Bootstrap internal validation with 1000 resamples showed mild optimism: the optimism-corrected AUC was 0.831, the calibration intercept was −0.02, the calibration slope was 0.88, and the Brier score was 0.142. The bias-corrected calibration curve showed acceptable overall agreement between predicted and observed probabilities, although some underestimation was observed at intermediate predicted probabilities and some overestimation was observed at higher predicted probabilities (Figure 5B).

**Figure 5:**
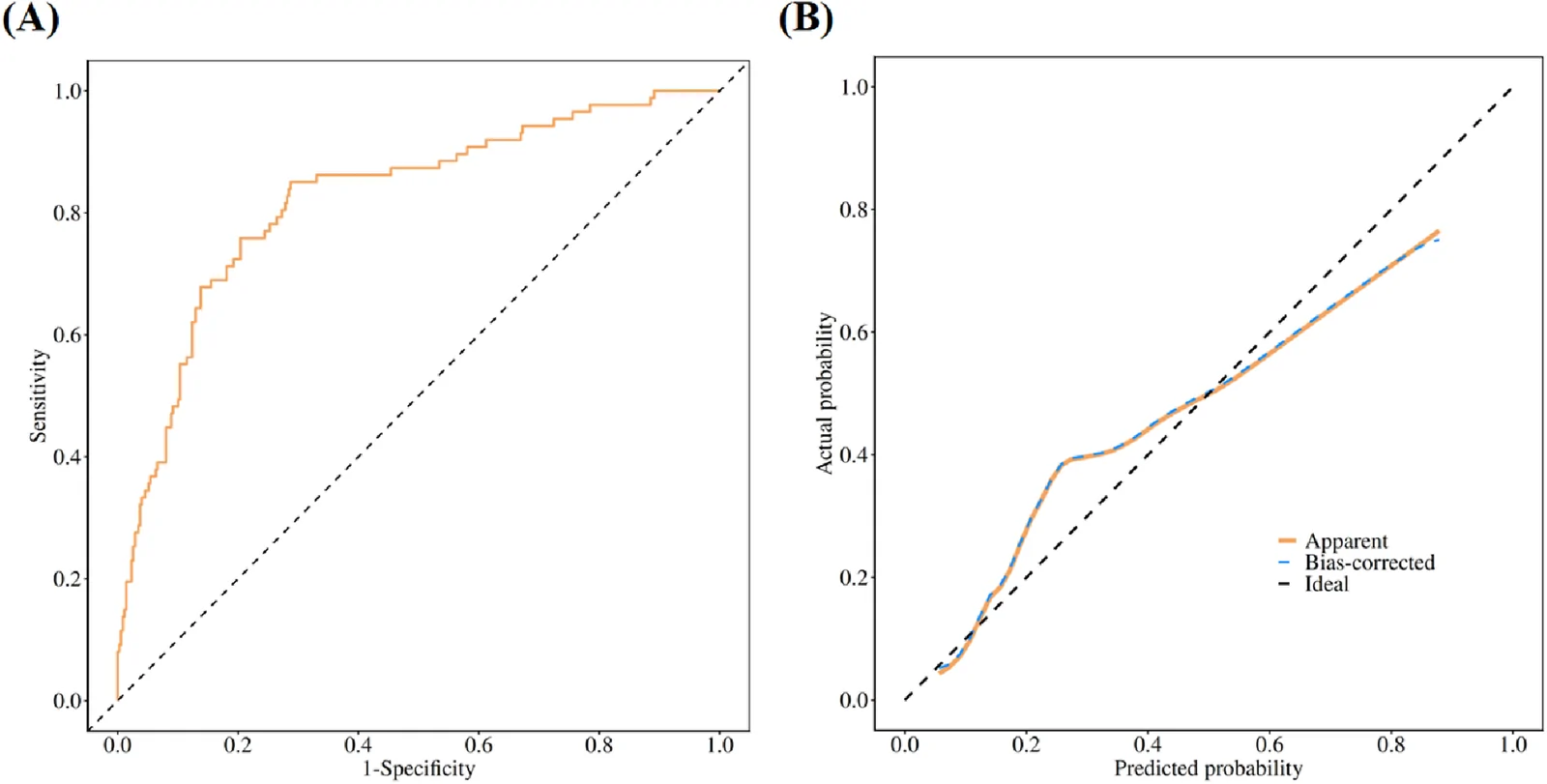
Apparent performance of the prediction-oriented MDRO isolation nomogram. **(A)** ROC curve for the prediction-oriented nomogram, which includes diabetes, invasive mechanical ventilation, combined antimicrobial therapy, and GCS score; length of RICU stay was excluded. **(B)** Bias-corrected calibration curve after bootstrap internal validation with 1000 resamples. No decision curve analysis is presented.

## 4 Discussion

According to this study, 37.25% (38/102) of culture-positive patients and 32.48% (38/117) of all enrolled patients had MDRO isolation. At the isolate level, Gram-negative bacteria made up 75.31% of all multidrug-resistant isolates. Klebsiella pneumoniae, Pseudomonas aeruginosa, Acinetobacter baumannii, Escherichia coli, and other dangerous bacteria were among them. Staphylococcus aureus, Staphylococcus epidermidis, Enterococcus faecalis, Streptococcus pneumoniae, and other Gram-positive multidrug-resistant bacteria made up 24.69% of the total. This finding is consistent with that of Macesic et al.'s study, which shows that the primary clinical problem in severe lung infections is multidrug-resistant Gram-negative bacteria. This is mainly due to the fact that Klebsiella pneumoniae, Pseudomonas aeruginosa, and Acinetobacter baumannii are prone to producing various inactivating enzymes, efflux proteins, and forming biofilms. The ventilator tubing and the RICU environment may serve as ongoing reservoirs for these organisms. Airway invasion operations can disrupt the natural barrier of the respiratory tract, significantly increasing the probability of colonization and invasive infection.

According to this study, patients in the MDRO group had worse clinical condition than those in the non-MDRO group, as well as lower GCS scores and longer RICU stays. This outcome is in line with the findings of an earlier investigation (13), indicating that MDRO isolation was associated with worse clinical condition with infectious pneumonia, prolongs the monitoring period, and increases the risk of adverse outcomes. This is mainly attributed to the resistance of multidrug-resistant bacteria to conventional antibiotics, poor initial anti-infection efficacy, continuous pulmonary infection triggering severe systemic inflammatory responses in patients with impaired spontaneous expectoration ability, further accumulation of sputum leading to increased infection, forming a vicious cycle, and increasing the risk of multiple organ damage and death (14, 15).

In addition, the study also found that diabetes mellitus, invasive mechanical ventilation, combined antimicrobial therapy, and length of RICU stay were factors associated with MDRO isolation. A higher GCS score was associated with lower odds of MDRO isolation, although this may reflect less severe illness, less sedation, or better airway protection rather than a direct protective effect. This result is similar to that of the study (16), and suggests that host immune deficiency, invasive airway procedures, antimicrobial exposure, and prolonged exposure to intensive care may be associated with multidrug-resistant bacterial infections. This is mainly because hyperglycemia inhibits the phagocytic and bactericidal functions of immune cells, hypoproteinemia damages the airway mucosal barrier and reduces the effective drug concentration in the body, invasive ventilation bypasses the oropharyngeal defense barrier, multiple antibiotics used in combination disrupt the respiratory tract microecology and screen out drug-resistant strains, length of RICU stay continuously increases the opportunities for cross-exposure to drug-resistant bacteria, and patients with a higher GCS score may have better airway protection and may be better able to drain sputum, reducing the colonization of pathogenic bacteria (17, 18). However, because this was a retrospective study, we could not determine whether prolonged RICU stay and combined antimicrobial therapy preceded or followed MDRO detection. These variables should therefore be interpreted as factors associated with MDRO isolation rather than causal risk factors. GCS may also reflect sedation, intubation status, and overall illness severity rather than acting as an independent factor associated with lower odds of MDRO isolation.

Additionally, the study discovered that there was no substantial multicollinearity across the variables because the VIF for any of the risk factors considered was less than 5. This result is similar to the conclusions of previous clinical risk regression studies, suggesting that the selected factors associated with MDRO isolation have good independence and may be useful for identifying patients at higher odds of MDRO isolation in clinical settings. This is mainly due to the fact that indicators such as diabetes, and mechanical ventilation respectively reflect the risk of infection from different dimensions (such as basic immunity, invasive procedures, and drug exposure), and there is no high-level overlap of correlations among the variables (19, 20).

On the basis of the factors associated with MDRO isolation, this study further built an exploratory nomogram to visualize factors associated with MDRO isolation in infectious pneumonia patients. Unlike complex regression equations, the nomogram converts each factor into a corresponding scoring scale; clinicians could calculate the total score and obtain an estimated probability of MDRO isolation. However, this approach remains exploratory and has not been prospectively evaluated. The study assessed the apparent performance of this nomogram in terms of discrimination and calibration. The ROC curve analysis showed apparent discriminative ability to distinguish patients with and without MDRO isolation; The calibration curve showed acceptable overall agreement between predicted and observed probabilities, although some underestimation was observed at intermediate predicted probabilities and some overestimation was observed at higher predicted probabilities. However, these findings are exploratory and require prospective validation. The prediction-oriented nomogram excludes length of RICU stay because it is known only after the RICU course. Length of RICU stay remains a clinical association variable in the multivariable model (Table 4). The nomogram includes diabetes, invasive mechanical ventilation, combined antimicrobial therapy, and GCS score. The main value of this study is the identification of factors associated with MDRO isolation. For patients with high scores, the intended response is not automatic escalation of antibiotics, but microbiological evaluation, review of previous cultures, and strengthening of infection-prevention measures. Because the prediction-oriented nomogram has not been prospectively validated, it cannot be used for early clinical decisions. Prospective multicentre external validation and clinical-impact assessment are required before bedside use.

**Table 4:** Multivariable logistic regression analysis of factors associated with MDRO isolation (aOR).

Indicators	Multivariable logistic regression analysis					
	*β*	SE	Wald χ^2^	aOR	95% CI	P
Intercept	1.711	0.353				
Diabetes	0.751	0.352	4.558	2.119	1.063–4.224	0.033
Invasive mechanical ventilation	1.047	0.379	7.642	2.849	1.357–5.980	0.006
Combined antimicrobial therapy	0.913	0.364	6.301	2.492	1.220–5.091	0.012
length of RICU stay	0.128	0.045	8.097	1.137	1.040–1.243	0.004
GCS Score	−0.394	0.098	16.182	0.674	0.556–0.817	<0.001

The dependent variable was lower respiratory tract MDRO isolation (1 = yes, 0 = no). Diabetes, invasive mechanical ventilation, and combined antimicrobial therapy were coded as 1 = yes, 0 = no; GCS score and length of RICU stay were continuous. Intercept = 1.711 (SE = 0.353). LP = 1.711 + 0.751 × diabetes + 1.047 × invasive mechanical ventilation + 0.913 × combined antimicrobial therapy + 0.128 × length of RICU stay−0.394 × GCS score. Predicted probability = 1/(1 + exp(−LP)). This equation refers to the multivariable association model, not to the prediction-oriented nomogram in Figure 4.

In light of this study's findings, clinical practice may consider a stratified prevention and control strategy: early screening for resistant bacteria in the lower respiratory tract should be done for pneumonia patients who have several high-risk characteristics at the same time; the duration of invasive mechanical ventilation should be strictly controlled, and airway care should be standardized; the hierarchical management of antibacterial drugs should be implemented to avoid unnecessary combined use of drugs, and the treatment should be adjusted in a stepwise manner according to drug sensitivity test findings; active control of blood sugar, and improvement of the body's immune function should be carried out; hand hygiene, environmental disinfection in the RICU, and single-room isolation for MDRO patients should be strengthened to reduce cross-transmission within the hospital. This study has several limitations. First, it was a retrospective single-centre study with a small sample size, incomplete susceptibility data, and an inability to completely distinguish colonization from true infection; intrinsic resistance was not specifically excluded when defining MDR. The primary outcome was therefore defined as lower respiratory tract MDRO isolation. Selection bias may have been introduced by excluding patients with severe multiple organ failure or malignancy, and the specific indication for radiotherapy was not available in the retrospective database. In addition, culture-negative patients are not a uniform group and may include patients with viral pneumonia, prior antibiotic exposure, or inadequate specimen quality; this heterogeneity should be considered when interpreting the results. Second, the timing of some predictors relative to MDRO detection was uncertain, and pneumonia type was not stratified, so the nomogram should be regarded as an exploratory risk stratification tool rather than a causal or strictly early prediction model. In addition, the sample size was small. The multivariable association model contained five predictors (EPV ≈ 7.6); the prediction-oriented nomogram contained four predictors (EPV ≈ 9.5). Bootstrap internal validation was performed; the optimism-corrected AUC was lower than the apparent AUC, indicating mild overfitting. External validation is still needed. The lack of standardized timing may introduce comparability bias between groups. Third, several potential confounders, including previous antimicrobial exposure, recent hospitalization, transfer from another healthcare facility, previous MDRO colonization or infection, chronic respiratory disease, immunosuppressive treatment, duration of ventilation and device use, and APACHE II or SOFA scores, were not available; residual confounding cannot be excluded, and GCS alone may not fully capture illness severity. Future prospective studies with predefined time points and standardized susceptibility testing are needed.

## 5 Conclusion

The clinical data of 117 infectious pneumonia patients at Tianjin Chest Hospital was retrospectively examined in this article. We found that the MDRO isolation rate was 37.25% (38/102) among culture-positive patients and 32.48% (38/117) among all enrolled patients. Klebsiella pneumoniae, Pseudomonas aeruginosa, Acinetobacter baumannii, and other Gram-negative bacteria are the most common resistant pathogenic microorganisms. MDRO isolation was associated with prolonged RICU stay and worse clinical condition. Through single and multiple factor regression, diabetes, invasive mechanical ventilation, combined antimicrobial therapy, and length of RICU stay were identified as factors associated with MDRO isolation. A high GCS score is associated with lower odds of isolation. A prediction-oriented nomogram was then constructed after excluding length of RICU stay, and it includes diabetes, invasive mechanical ventilation, combined antimicrobial therapy, and GCS score. The main value of this study lies in the exploration of factors associated with MDRO isolation rather than in promoting the nomogram for widespread clinical use. The model has not been prospectively evaluated and should not be assumed to improve empirical antimicrobial selection, antimicrobial stewardship, infection prevention, or patient outcomes. Prospective multicentre external validation, followed by clinical-impact assessment, is necessary before it can be recommended for bedside use.

## Data Availability

The original contributions presented in the study are included in the article/Supplementary Material, further inquiries can be directed to the corresponding authors.
